# Efficacy of atomoxetine and oxybutynin in the treatment of pediatric obstructive sleep apnea, a three case report

**DOI:** 10.3389/frsle.2025.1682625

**Published:** 2025-11-04

**Authors:** Helena Larramona Carrera

**Affiliations:** Department of Pediatrics, Unit of Pediatric Pulmonology, Hospital Universitari Parc Tauli, Sabadell, Spain

**Keywords:** obstructive, sleep, apnea, pediatric, pharmacotherapy

## Abstract

Persistent severe obstructive sleep apnea (OSA) after adenotonsillectomy (AT) is not uncommon in children with genetic syndromes and/or obesity. Although continuous positive airway pressure (CPAP) is the standard treatment, adherence in pediatric patients is often low, limiting its effectiveness. We report three cases of children with persistent OSA and failure to continue CPAP therapy, in whom an alternative pharmacological approach was explored. In agreement with their families, a 4-week trial of combined atomoxetine and oxybutynin was initiated. Of note, the first patient was concurrently treated with lisdexamfetamine for attention deficit hyperactivity disorder, while the second had morbid obesity under treatment with liraglutide. Both patients demonstrated a great improvement in their apnea-hypopnea index (AHI), with reductions >50% measured by polysomnography. The combination therapy was well tolerated, with no significant adverse effects or interactions with ongoing medications. The third patient did not adhere to the drug therapy, and the effect of a single night of treatment before the follow-up polysomnography was evaluated, showing no change in AHI. These cases suggest a potential role for atomoxetine and oxybutynin as alternative therapeutic options for pediatric OSA in complex scenarios where severe OSA persists despite AT and failed CPAP therapy, warranting further evaluation in large pediatric clinical trials. Nevertheless, the final case underscores that even pharmacological treatments, although seemingly straightforward to administer, may encounter adherence challenges.

## Introduction

The persistence of severe Obstructive Sleep Apnea (OSA) after adenotonsillectomy (AT) in children with genetic disorders and/or obesity is not uncommon. The effectiveness of continuous positive airway pressure (CPAP) devices in the pediatric population is limited, mainly due to poor adherence. A retrospective U.S.-based big-data study evaluated CPAP adherence in over 20,000 pediatric OSA patients using data uploaded nightly from a brand specific device linked to a single insurance provider (Bhattacharjee et al., 2020). After 90 days, 61.8% of patients remained on therapy without discontinuation, yet only 46.3% met the recommended adherence of at least 4 h per night on 70% of nights. A recent meta-analysis including over 22,000 children with OSA reported that only 46.56% maintained CPAP use for at least 4 h nightly (Sawunyavisuth et al., 2023). Both studies revealed that adherence to CPAP therapy remains suboptimal (<50%), and reveals the persistent challenge of maintaining long-term compliance in pediatric OSA management.

Pediatric OSA has been associated with cognitive deficits, behavioral disorders such as hyperactivity and inattention, and cardiovascular complications, among others. Of note, disrupted nocturnal blood pressure regulation and early signs of cardiac dysfunction, especially in more severe cases have recently found (Witter et al., 2025). Addressing OSA promptly may lower the risk of developing hypertension and long-term cardiovascular complications in short and long-term. A population-based analysis in children and adolescents estimated that sleep-disordered breathing (SDB) increases three times the incidence of developing neurobehavioral problems, and being more prevalent SDB in children with developmental impairment (Zhang et al., 2024). Furthermore, OSA triggers persistent systemic inflammation, and experimental models of intermittent hypoxia have demonstrated elevated inflammatory markers across blood and vital organs (Koritala et al., 2024). Given the association of OSA with neurocognitive and cardiovascular complications and a state of persistent systemic inflammation, there is a pressing need to explore effective and easy-to-implement alternative therapies.

The combination of atomoxetine and oxybutynin is an emerging possible treatment in OSA in adults and children with Down syndrome. The combination of noradrenergic and antimuscarinic agents may enhance upper airway muscle tone during sleep, thereby reducing pharyngeal collapse (Chan et al., 2006; Grace et al., 2013). In 2023 a meta-analysis including eight randomized controlled trials found that therapy combining noradrenergic and antimuscarinic drugs produced significant reductions in the AHI (−9.03 events/h, *p* = 0.01) and increases in minimum oxygen saturation (5.61%, *p* < 0.01) compared with placebo (Lee et al., 2023). In 2024, another meta-analysis including 13 randomized controlled trials involving 345 participants demonstrated that the combined pharmacological regimen significantly reduced the AHI [−6.30 events/h, *p* = 0.0003], and improved most gasometric parameters, specifically, improvements were observed in hypoxic burden, oxygen desaturation index 3% and 4%, and nadir oxygen saturation (Bady et al., 2024). In the first pediatric randomized crossover trial including 11 children with Down syndrome and OSA, they found that both low- and high-dose atomoxetine–oxybutynin produced similar effects, with about a 50% reduction in AHI after 1 month of treatment (Combs et al., 2023).

We report in this manuscript an improvement of OSA severity with a greater reduction of 50% of AHI in two patients receiving atomoxetine and oxybutynin for 1 month and no changes in a patient with low adherence of drug therapy but the night of control polysomnography. Polysomnography parameters were manually scored according to the most recent American Academy of Sleep Medicine pediatric scoring criteria, consistently performed by the same pediatric pulmonologist with expertise in pediatric sleep scoring.

## Case 1

A 10-year-old girl with Autism Spectrum Disorder due to AUTS2 deficiency had severe persistent OSA after AT. After several attempts to adapt to CPAP with failure in its continuous use (<1 h), it was decided in agreement with the family to explore treatment with atomoxetine and oxybutynin for 4 weeks. Her Body Mass Index (BMI) is 26.1 kg/m^2^. Patient received 25 mg atomoxetine and 5 mg oxybutynin in the 1st week, and after a call phone to assure adherence and tolerability, 60 mg atomoxetine and 5 mg oxybutynin. The patient was already being treated with lisdexamfetamine for Attention Deficit Hyperactivity Disorder (ADHD).

Drug-induced sleep endoscopy showed obstruction of the anteroposterior palatine velum, septal dysmorphism, bulky tongue base and no adenoids.

There was a great improvement in the Apnea and Hypopnea Index (AHI) measured with polysomnography with a reduction of 72% (AHI 30.4 events/h to an AHI of 8.6 events/h) and desaturation levels with nadir from 73% to 86% (Table 1). No changes in sleep architecture were noticed, with a similar percentage of REM sleep (12.9% and 13.1%).

**Table 1:** Polysomnography variables baseline and after drug therapy.

	**Case 1**		**Case 2**		**Case 3**	
Age, y	10		14		15	
BMI, kg/m^2^	26.1		31.2		44.5	
	Pre-treatment	Ato-Oxy 28 days	Pre-treatment	Ato-Oxy 28 days	Pretreatment	Ato-Oxy Night PSG
AHI events/h	30.4	8.6	7.1	3.3	31.5	45.3
AHI NREM events/h	29.1	4.9	0.6	3	26	49.1
AHI REM events/h	38.9	33.8	31.3	7.5	48.8	22.7
Time with SpO_2_ <90%	0.9%	0.1%	0.3%	0%	2.9%	0.6%
Nadir	73%	86%	87%	90%	84%	86%
Hypoxic burden^*^ %min/h	27.1	15.4	21.5	2	61.3	73.7
REM sleep	12.9%	13.1%	21.3%	5.5%	23.5%	16.4%
N1	22.3%	18.3%	3%	1.9%	6.8%	6.1%
N2	33.2%	36.3%	41.8%	54.3%	42.2%	54.3%
N3	31.6%	32.3%	33.9%	38.4%	27.5%	23.2%
Efficiency	63.1%	73.5%	93.3%	86%	97.1%	67.5%
Total Sleep Time, minutes	286.3	298.5	405.3	293	434	338
Sleep latency, minutes	26.5	16.5	0	11	5.5	26.5
Wake After Sleep Onset, minutes	141	90.9	29	36.5	7.4	135.9

^*^Hypoxic burden: sum of the area under the desaturation curve for each apnea or hypopnea event and dividing the total by the total sleep time.

Treatment was well tolerated with several days of self-limited abdominal discomfort and no interactions were observed with his previous treatment with lisdexamfetamine.

## Case 2

A 14-year-old adolescent girl with obesity (BMI 31.2 kg/m^2^) and asthma presented with persistent OSA following AT. Preoperative polysomnography revealed prolonged oxygen saturations not optimal (90–93%) during REM sleep, poorly controlled asthma, and antihypertensive medication use. After AT, antihypertensive therapy was no longer required, but her obesity worsened, and asthma control remained suboptimal. CPAP therapy was initially well tolerated during the first year but later became intermittent, and the patient eventually discontinued use. As her BMI increased to 38 kg/m^2^, liraglutide treatment was introduced, resulting in a BMI reduction of −7 kg/m^2^; however, she subsequently discontinued both liraglutide and CPAP, and no further weight loss was achieved. In agreement with the family, a 4-week trial of atomoxetine and oxybutynin was initiated.

During the first week, she received atomoxetine 25 mg and oxybutynin 5 mg, followed by 80 mg atomoxetine with 5 mg oxybutynin after tolerability was confirmed through follow-up calls. Treatment was generally well tolerated, with transient abdominal pain and a single episode of vomiting in the first week. Polysomnography demonstrated a marked improvement, with AHI reduced by over 50% with an AHI below 5 events/h (Table 1). REM sleep duration was significantly reduced compared to baseline, although overall sleep efficiency was good. The patient reported no insomnia or difficulty maintaining sleep at home, and her school performance improved.

## Case 3

A 15-year-old adolescent girl with morbid obesity (BMI 44.5 kg/m^2^) presented with severe persistent OSA following AT, with an AHI of 69 events/h and an oxygen nadir of 77%. Polysomnography at age 11 had led to the initiation of CPAP therapy, but usage remained intermittent in the first 6 months (<4 h/night) and ultimately dropped to zero over the last 3 months despite multiple attempts to address adherence issues. In addition, awake endoscopy showed no regrowth of adenoids and no tonsils. Given the benefits observed in the previous two cases, a 4-week trial of atomoxetine and oxybutynin was undertaken in agreement with the patient and her family, with baseline polysomnography showing an AHI of 31.5 events/h.

Initial dosing consisted of atomoxetine 25 mg and oxybutynin 5 mg during the first week. Despite repeated calls to assess adherence and tolerability, no response was obtained, and the day before the follow-up polysomnography, the family confirmed non-adherence. Due to efficacy with one night dose in adult studies, atomoxetine 80 mg and oxybutynin 5 mg were administered prior to polysomnography to evaluate efficacy. No overall improvement in AHI was observed, except for reductions in AHI during REM sleep (Table 1). Sleep efficiency decreased, and REM duration was shorter compared with the baseline study. The family reported difficulty ensuring that the patient consistently took the medication (Azarbarzin et al., 2019).

## Discussion

The main findings are that 1 month of treatment with atomoxetine and oxybutynin in these children reduced OSA severity, as reflected by changes in the AHI, and improved overnight nadir oxygen saturation. Moreover, no significant changes in sleep efficiency were observed and the regimen was well tolerated with no interactions with other medications. However, one case was not adherent to the drug regimen and no efficacy was observed with one night treatment. Thus, even pharmacotherapy given once at night could be a challenge for some patients.

### Effects on OSA severity

Adult studies of noradrenergic–antimuscarinic pharmacotherapy show statistically robust but clinically moderate effects on OSA severity. Nevertheless, in pediatric patients, where lower AHI cutoffs define severe disease, even small reductions may translate into significant benefit. The association of neurocognitive and cardiovascular disorders with OSA makes it necessary to explore new effective and easy-to-implement therapies. Take into account that AHI is not the only parameter to consider in OSA severity; it is relevant for assessing treatment efficacy, along with the marked reduction in hypoxic burden in Cases 1 and 2, and the improvement in nadir and time spent with saturations below 90% in all three cases.

In adult studies a high dose of atomoxetine (80 mg) showed more efficacy than lower dose (40 mg) being same standard dose for oxybutynin (5 mg). Even, a recent study demonstrated the combination of atomoxetine with a non-specific antimuscarinic such as oxybutynin (broad M-subtype receptor selectivity) had better results than more selectivity muscarinic receptor (M2 and M3 or M1 muscarinic receptor selectivity; Aishah et al., 2021).

In the pediatric study by Combs, two doses of atomoxetine were investigated, low dose (0.5 mg/kg, maximum 40 mg) and high dose (1.2 mg/kg, maximum 80 mg) with same doses for oxybutynin (5 mg) and similar effect were found with an approximately 50% reduction in AHI (Combs et al., 2023). In the three cases, it was decided to approach a high dose due to studies in adults support a high dose in severe cases (Rosenberg et al., 2022). In the pediatric trial, the mean AHI was 7.4 events/h while in these three cases AHI were in the spectrum of severe pediatric OSA.

### Rationale to explore in children

In the first study by Taranto-Montemurro, the combination of atomoxetine and oxybutynin obtained an AHI mean difference of −20 events/h in the group treated with a 63% reduction of AHI (Taranto-Montemurro et al., 2019). Subsequent studies in adults with combination of different regimens with a norepinephrine reuptake inhibitor and an antimuscarinic have been showed variable reductions in AHI. The heterogeneity of the results may be related to different predominance of physiological factors causing OSA (upper airway collapsibility, loop gain, arousal threshold and upper muscle responsiveness) among others methodological factors. In a physiological study, it was found notable improvements in upper airway stability, enhanced breathing control, and a modest reduction in arousal threshold (Taranto-Montemurro et al., 2020). Individuals with milder airway collapsibility and lower baseline AHI were most likely to achieve OSA resolution with atomoxetine–oxybutynin. Children typically present with a mildly collapsible pharyngeal airway, an endotype expected to result in greater therapeutic benefit. However, larger studies are needed to find the patient population who would benefit most from this drug combination for the management of OSA weighing the risks vs. benefits of using this therapy long term. Although atomoxetine appears to play the primary role in reducing respiratory events, it is not effective as monotherapy. Moreover, oxybutynin may mitigate the wake-promoting effects of atomoxetine due to its sedative properties.

Endogenous norepinephrine withdrawal during NREM sleep and inhibitory effects of active muscarinic receptors during REM sleep are crucial in the reduction of pharyngeal dilator muscle during sleep (Chan et al., 2006; Grace et al., 2013). Atomoxetine prevents norepinephrine from being reabsorbed by the hypoglossal motor neurons increasing its level in NREM sleep. Oxybutynin blocks acetylcholine receptors on hypoglossal motor neurons (an inhibitory muscarinic process), making the genioglossus muscle more responsive in REM sleep. However, current evidence indicates that reduced activity of upper airway muscles during REM sleep is mediated by both pathways, noradrenergic and cholinergic mechanisms (Taranto-Montemurro et al., 2023). In addition, the synergistic effect of atomoxetine and oxybutynin highlights oxybutynin's role in improving upper airway collapsibility during NREM sleep (Taranto-Montemurro et al., 2023). Therefore, reducing the AHI appears to require combining atomoxetine with oxybutynin, probably due to enhanced activity of the genioglossus muscle across sleep stages. Although the underlying mechanism is not yet fully understood, the synergistic effect of adding an antimuscarinic to the noradrenergic agent seems essential for the therapeutic benefit observed. In addition, the combination has been shown to reduce arousal threshold. In these cases, Case 1 demonstrated a marked reduction in AHI during NREM sleep but not in REM, whereas in Cases 2 and 3, improvements were observed only in REM AHI; however, due to the limited amount of REM sleep in Case 2, it is difficult to determine whether the reduction in REM AHI was genuine. Nevertheless, in all three cases, as in most pediatric patients, baseline polysomnography was predominantly characterized by REM-related OSA. Understanding why REM AHI or NREM AHI improves in some cases but not others would warrant further evaluation in a larger cohort of children with severe OSA.

### Adverse effects

The adverse effects reported in Cases 1 and 2 were self-limited abdominal discomfort. Both agents hold individual medicaments agency approval for pediatric use—atomoxetine for managing ADHD and oxybutynin for treating overactive bladder. Reports in their use separately in children show good tolerance.

In the crossover trial of children with Down syndrome treated with both low and high doses of atomoxetine–oxybutynin, irritability and fatigue were the most frequently reported side effects, generally presenting with mild intensity (Combs et al., 2023). Another side effects were diarrhea, headaches, abdominal pain, decreased urinary frequency and dry mouth. The group of high doses explained more adverse effects.

There have been studies that show that atomoxetine can have a risk of suicidal ideation in children. All patients underwent psychiatric and/or psychological evaluation prior to initiation, and atomoxetine treatment was deemed appropriate. A telephone follow-up specifically inquired about changes in mood, behavior, or any parental concerns.

In adult patients, a short regimen of the combined therapy is mainly associated with mild effects such as elevated heart rate, dry mouth, and occasional difficulty with urination. Thus, heart rate during sleep increases by about 10% in most studies, yet morning blood pressure remained unchanged. In the pediatric study by Combs, no significant changes in resting heart rate or increases in blood pressure were observed. Notably, while children initiating atomoxetine for ADHD typically exhibit an early increase in heart rate and blood pressure, these effects tend to stabilize and even decline with continued treatment over several months and no risk for serious cardiovascular events (Houghton et al., 2020).

Rapid escalation of atomoxetine dosage has been linked to greater adverse effects, suggesting that a more gradual titration, such as 1-week used in these patients following the pediatric trial, could help reduce side effects.

It is necessary to explore the long-term effects and their impact on clinical outcomes. To date, the longest treatment in these cases is 6 months, and no additional adverse effects have been reported, with good tolerance observed.

### Sleep variables

Sleep efficiency remained comparable between baseline polysomnography and treatment with atomoxetine–oxybutynin in Cases 1 and 2, but was markedly reduced in Case 3, likely due to the inability to titrate the dose as the patient was not taking the medication. REM sleep duration was unchanged in Case 1, whereas a substantial reduction was noted in Cases 2 and in less extent in Case 3. Of note, several studies have noted a decrease in the proportion of REM sleep. While this reduction might lessen vulnerability to REM-related OSA often seen in children, the possible long-term consequences remain uncertain.

In 2023, a meta-analysis did not observe changes in sleep efficiency. However, the combination of these drugs was associated with alterations in sleep architecture, including shortened REM sleep (−8.41%, *p* < 0.01; Lee et al., 2023). In a more recent meta-analysis, total sleep time remained unchanged, however alterations in sleep architecture and efficiency were observed compared with placebo (Bady et al., 2024). No significant sleep architecture differences were present with atomoxetine and oxybutynin in the pediatric trial (Combs et al., 2023).

### Limitations

A limitation of these case reports is the absence of clinically validated and quality-of-life questionnaires to assess treatment benefits. Instead, open-ended questions and discussions with families and patients were used to evaluate clinical outcomes. In fact, in the pediatric study by Combs, no significant differences in OSA-18 scores were observed (Combs et al., 2023). Another limitation is the effect of drugs respect heart rate was not evaluated as these patients were already treated with other medications potentially affecting heart rate. Adherence was not objectively measured due to the family's agreement to explore this new treatment. However, after the first week, tolerability, adverse effects, and adherence were monitored via phone calls. Moreover, the sleep technician asked the patients and observed that they took the medication as administered, 30 min before bedtime.

A possible explanation for the lack of efficacy in the third patient is that this adolescent was at a more advanced stage of hormonal development. It has been suggested that non-menopausal females may be less responsive to AHI reduction with atomoxetine–oxybutynin, although female representation in adult studies has been limited. Another explanation could be probably a greater upper airway collapsibility and only one dose night was not enough to show improvements.

## Conclusions

In two cases, the combination of atomoxetine and oxybutynin significantly reduced the severity of OSA and was well tolerated. Despite the limited number of pediatric cases with this new combination drug therapy, the results obtained are at least encouraging and offer more therapeutical options when adenotonsillectomy and CPAP fails. However, this combination could be difficult to implement in some patients.

Noradrenergic and antimuscarinic therapy shows potential for pediatric OSA management, but evidence on its long-term safety is lacking. Reported concerns include insomnia and cardiovascular effects in adults such as increased heart rate, highlighting the need for further research on sleep architecture and heart parameters.

## Patients' perspective

The patients in cases 1 and 2, along with their families, are pleased to have achieved clinical improvement and to no longer face the nightly struggle of ensuring CPAP use. The patient in case 3 did not verbally refuse treatment and initially agreed to try it; however, in practice, she did not adhere to the regimen. Her family felt unable to insist on nightly compliance, although they do not rule out attempting it in the future. All are grateful for the emergence of new, easier-to-implement therapies that are effective in resolving or improving OSA.

## Data Availability

The raw data supporting the conclusions of this article will be made available by the authors, without undue reservation.
